# *Caulerpa chemnitzia* in Darwin threatening Galapagos coral reefs

**DOI:** 10.1371/journal.pone.0272581

**Published:** 2022-08-31

**Authors:** Inti Keith, William Bensted-Smith, Stuart Banks, Jenifer Suarez, Bernhard Riegl

**Affiliations:** 1 Charles Darwin Research Station, Charles Darwin Foundation, Santa Cruz, Galapagos, Ecuador; 2 Galapagos National Park Directorate, Puerto Ayora, Galápagos, Ecuador; 3 National Coral Reef Institute, Halmos College of Arts and Sciences, Nova Southeastern University, Dania Beach, Florida, United States of America; California Academy of Sciences, UNITED STATES

## Abstract

Coral reefs are rare in the Galapagos and there is concern that, like in many areas around the world, they may be degrading due to increasing anthropogenic pressure, which can cause changes and reorganizations of structure and function with associated phase shifts. Algae of the genus *Caulerpa* J.V. Lamouroux, 1809 are known as widespread and persistent marine invaders. They grow rapidly, particularly in disturbed areas where they can opportunistically monopolize substratum and compete with native species, thus reducing biodiversity. *Caulerpa chemnitzia* increased in abundance and overgrew corals on the reef since 2012, ultimately raising fears that a phase-shift from coral to algae might be imminent. However, from 2019 onwards algae populations strongly contracted and while not having returned to baseline level, there is currently low risk of corals being displaced. Visual censuses were conducted on a yearly basis since 2004 using sample quadrats (0.5 x 0.5m) every 5 m along a 50-m-long transects at a depth of 6–15 m at 5 permanent subtidal ecological monitoring sites around Darwin. In addition, 10 m photo-transects were taken using a graduated meter-long measuring stick in the centre of the frame in 2012, 2014, 2016, 2017, 2018 and 2021 at a depth of 15m at Wellington reef. The authors hypothesize that this species could have expanded its distribution over Wellington Reef because of its known morphological plasticity due to a response to change in the environment, in this case high temperature and low nutrients. As ENSO events are predicted to increase in intensity and frequency due to the impact of climate change it is important to develop and implement a functional alert system. Early Detection Rapid Response (EDRR) protocols are recommended to avoid climate driven Non-Indigenous Species (NIS) entering the GMR or for native species becoming invasive due to warming-related phase shifts.

## Introduction

The Galapagos archipelago is located 1,000 km off the coast of Ecuador in the Eastern Tropical Pacific (ETP) and protected within the Galapagos Marine Reserve (GMR) which extends 40 nautical miles from the coastal baseline, an area of about 138,000 km^2^ [1–3]. Its globally unique marine biodiversity represents an unrivalled biological and environmental field laboratory [4]. Its geographic isolation has limited natural immigration of new species, historically enabling those few species that did arrive to evolve in the absence of competitors and predators [5]. However, the increase in human population and marine traffic has led to the introduction of alien species with many more regarded as cryptogenic species (i.e. not demonstrably native or introduced [6, 7]

Coral reefs are rare in the Galapagos [8] and there is concern that, like in many areas around the world, they may be degrading due to increasing anthropogenic pressure [9], which can cause changes and reorganizations of structure and function with associated phase shifts [10]. Algae of the genus *Caulerpa* J.V. Lamouroux, 1809 are known as widespread and persistent marine invaders [11, 12]. They grow rapidly, particularly in disturbed areas where they can opportunistically monopolize substratum and compete with native species, thus reducing biodiversity. This can even occur in their native ranges [13]. Their ability to monopolize space and alter food webs makes macroalgae particularly damaging marine invaders [12–16].

Galapagos algae have been studied since the 1831 Beagle expedition by several subsequent scientific expeditions [17–20]. Sponsored by Allan Hancock aboard the vessel Velero III in 1934 and 1939, Taylor described over 50 new algae [21]. Historically several *Caulerpa* species have been reported in different islands of the archipelago (*C*. *racemosa var*. *clarifera*, *C*. *racemosa var occidentalis*, *C*. *racemosa var*. *uvifera* and *C*. *peltata*) [17, 21, 22]. Subtidal Ecological Monitoring around the archipelago on a yearly basis since 1995 [2, 23–25] has revealed two hitherto unrecorded species of *Caulerpa* present in different locations around the archipelago, *Caulerpa chemnitzia* (Esper) Lamouroux, previously recorded as *Caulerpa peltata* [21, 26, 27] and *Caulerpa racemosa* also recorded as *Caulerpa racemosa var*. *clarifera* and *uvifera* (Forsskål) J. Agardh, [17, 21, 28–31]

*Caulerpa chemnitzia* [8, 32, 33] has been observed to exhibit rapid expansion at Wellington Reef, the only frame building coral reef in the archipelago [33]. If this trajectory were to continue, the algae’s presence and expansion could expose this biological important region to coral mortality and a potential shift towards algal dominance. This present study provides a characterization of the expansion of *C*. *chemnitzia* on Darwin Island’s Wellington Reef by documenting the abundance of algae on the reef over the past 15 years and discussing possible scenarios for the observed population outbreak and future impacts on the biodiversity of this important reef system in scenarios involving introduced species and climate change.

## Methods

### Study site

**Fig 1:**
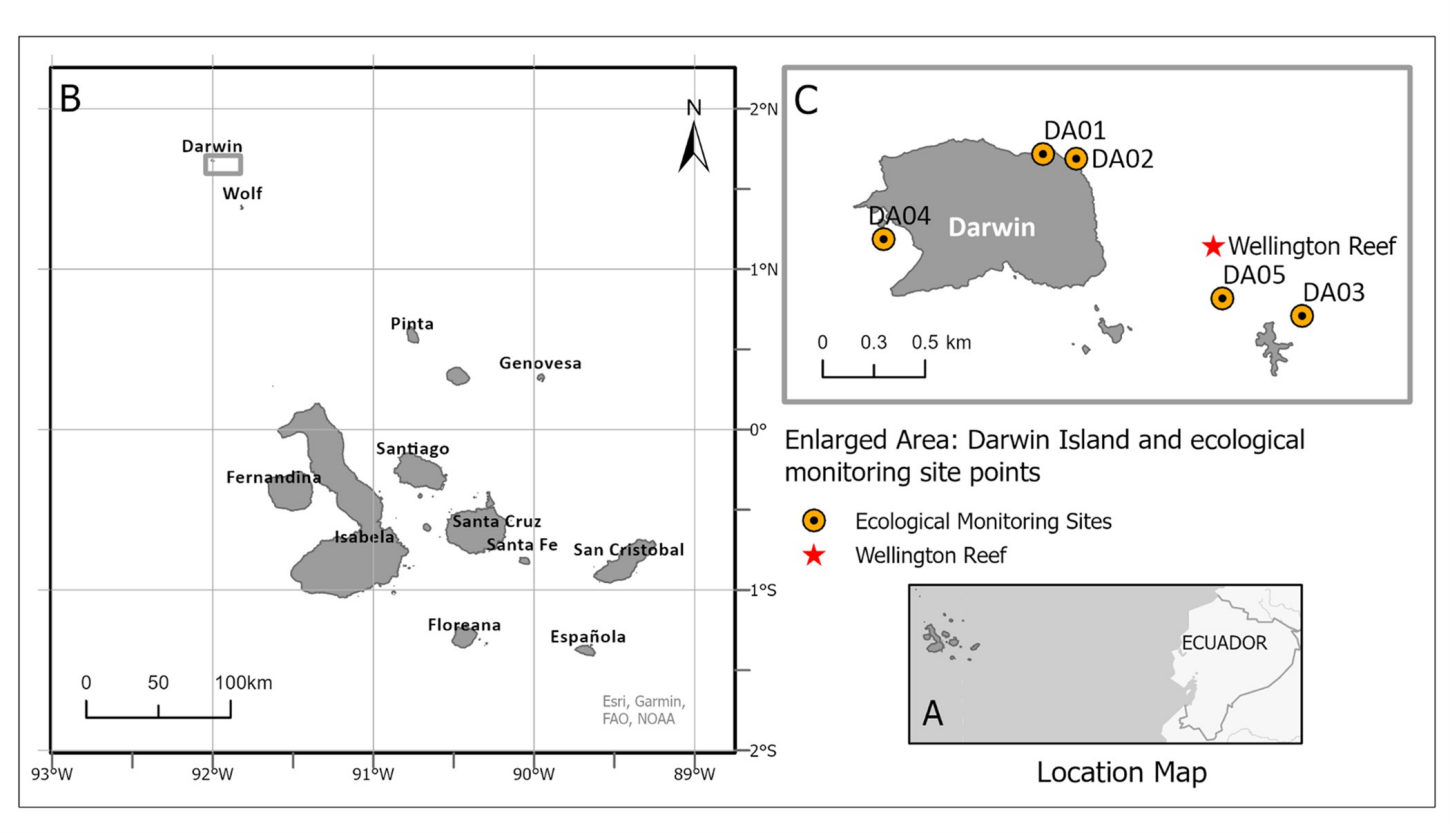
Location of *Caulerpa chemnitzia* study. (A) The **Galapagos Islands** are an archipelago off the coast of Ecuador (B) an overview of all islands, highlighting Darwin the northernmost island (C) 5 subtidal ecological monitoring sites and, Wellington reef, the site used for the collection of photo-transects data.

The Galapagos Islands are in the Eastern Tropical Pacific (ETP) 1000km off the coast of Ecuador. The islands of Darwin and Wolf are the most northern islands in the Galapagos archipelago and are situated within the Galapagos Marine Reserve (GMR) (Fig 1A and 1B), forming the Far North biogeographical region [34]. This area has the highest cover of reef-building corals and is regionally important for connectivity and persistence of corals in the Eastern Tropical Pacific [33]. The confluence of warm and cold currents in Galapagos allows for unique biological communities [35, 36]. Different water masses, current systems, high levels of productivity, diversity of ecosystems and natural connectivity exist due to the convergence of major currents. [4, 37–39]. Darwin and Wolf are situated outside the main upwelling area and are influenced by the Panama Current that brings warmer waters from the northeast, making this region the warmest and most tropical of the archipelago [8, 37, 40]. The present paper focusses on sites around the island of Darwin and Wellington Reef the only true framework reef in Galapagos [41], which is situated between the former Arch (which collapsed in 2021) and the island proper, on a north-facing shallow shelf caused by wave abrasion of the largely tuffaceous material (Fig 1C).

### Data collection

There has been an ongoing subtidal ecological monitoring effort since 1995, with the currently employed standard method for reef communities introduced in 2004 [42]. Five permanent subtidal ecological monitoring sites exist around Darwin and visual censuses were conducted along 50-m-long transects at a depth of 6–15 m. These stations are Darwin Anchorage Point North (DA01), Darwin Anchorage Point South (DA02), Darwin’s Arch (DA03), the Hidden Reef (DA04) and Wellington Reef (DA05) (Fig 1C). Sample quadrats (0.5 x 0.5m) were placed systematically every 5 m along the 50 m transect, each quadrat was made up by a 5 x 5 cm grid system constructed with polypropylene twine creating 81 intersection points. In each quadrat, all species that fell in the 81 intersections were counted and recorded. Species that did not fall in the intersections were recorded as present. In addition 10 m photo-transects were taken using a graduated meter-long measuring stick in the centre of the frame in 2012, 2014, 2016, 2017, 2018 and 2021. These photo-transects were taken at a depth of 15m at the site known as Wellington reef (Fig 1C)

### Data analysis

**Fig 2:**
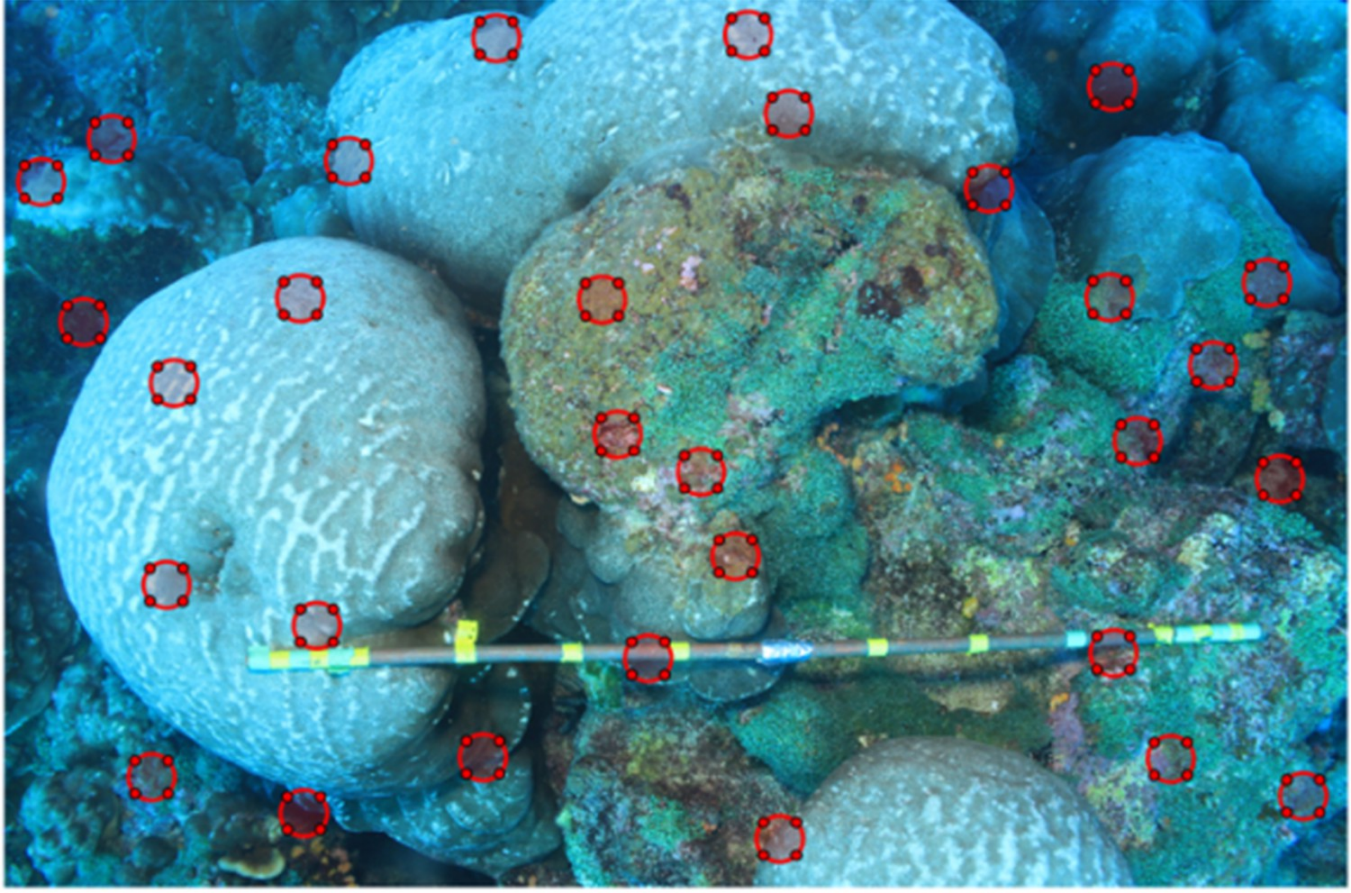
Coral with regions of interest (ROIs). Example of method used to determine percentage cover of *C*. *chemnitzia*, using circular regions of interest (ROIs), shown by the red circles on the image.

To analyse the change in coverage of *C*. *chemnitzia* for the photo-transects, 30 regions of interest (ROI) were plotted within each quadrat photograph. ROI sizes were chosen to be equivalent of ~50 cm in pixels (Fig 2). Pixel diameters had to be modified by reprocessing the images each year due to changing image resolutions. Once the resolution of the image was considered, 30 random permutations were taken within the *x* and *y* pixel ranges of the image to be the centre of the ROI. The pixel range excluded the pixel equivalent of 1.25 radii of the ROI from the end point of the *x* and *y* axes in order to avoid the ROI being created partially outside the top and right edges of the image. To prevent the same issue occurring on the bottom and left edges, a logical test was performed to verify if any of the random permutations fell below the pixel equivalent of 1.25 radii of the ROI, if this was the case new sets of 30 permutations were created until one passed the test. The following step ensured that there were no overlaps between the ROI by comparing the hypotenuse distances between the centre point of each and ensuring it was greater than 4 radii, otherwise the 30 permutations would be chosen again. This also made it more likely that the ROI were better spaced out across the image. For all 30 ROI to be plotted, the locations of each had to pass the two before mentioned logical tests. The MATLAB script used is provided in (S1 File).

The chosen diameter allowed for relatively easy assignment of each ROI to 5 separate categories depending on the cover of *C*. *chemnitzia*: 100% full, ~75% full, ~50% full, ~25% full, and containing no traces of the alga. This allowed for a more accurate depiction of the percentage coverage of *C*. *chemnitzia* across the whole transect. These average percentage cover of the macroalgae for each transect was then plotted to observe the trend of *C*. *chemnitzia* growth across the years. The same method was also performed for hermatypic coral coverage across the range of images. The species of coral observed in the images were *Porites lobata*, *Pavona gigantea*, *Pavona clavus*, *Pavona chiriquiensis* and *Pocillopora sp*., but there are known to be other species of hermatypic corals present in the region such as *Pavona varians* and *Pavona maldivensis* [43]. Coral cover was found to vary occasionally across the years due to the photo-transects not being taken along a fixed transect. Therefore, to be able to better visualise the cover of *C*. *chemnitzia* in relation to that of hermatypic corals, each of the average percentage coverages per transect was multiplied by a factor so the value for hermatypic corals was equal to one, so as to visualise the ratio of C. *chemnitzia* coverage to that of the corals. The following assumptions were made for the data collection within the analysis: Firstly, that the real-life size of the ROI was constant across the whole image, it was also assumed that the height of the camera above the seabed and thereby the size of the image on the seafloor was constant across all images.

### Temperature data

**Fig 3:**
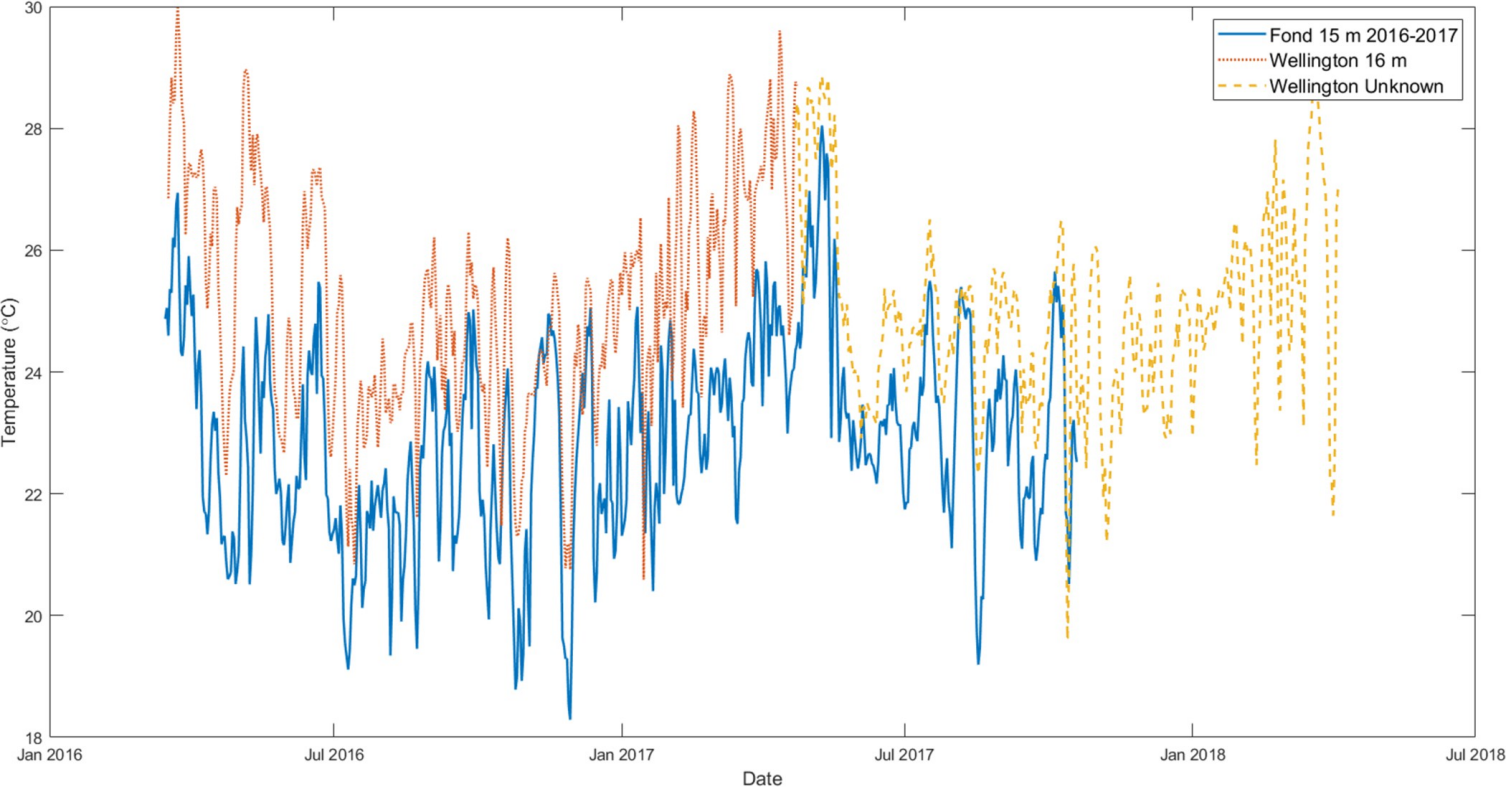
Daily temperature average from Wolf Island and Darwin Island. Comparison between the daily averaged logger temperature data at Wolf Island’s anchorage point (blue solid line), and loggers at Darwin’s Wellington reef at 16m (orange dotted line) and an unknown depth assumed to be around 15 m (yellow dashed line).

Temperature data were compiled from loggers placed at the anchorage point of Wolf Island. This is the most consistent site at which loggers have been placed since 2011, with only one period of close to 10 months of missing temperature data, thus covering the examined monitoring period. Loggers were always placed at the same depth (20 m) making accurate data comparison possible (one logger from 15-3-2016 to the 28-4-2017 was placed at 15 m). An issue with taking temperature data from loggers located at Wolf is that they will likely not exactly replicate the temperature fluctuations and levels from Darwin’s Wellington Reef, from where biological monitoring data for this study were obtained. But only a limited number of loggers had been placed at Darwin over the years and then at varying depths. To verify that temperature data from Wolf would be valid when analysing *C*. *chemnitzia* and hermatypic coral levels in Darwin, three sets of logger data were compared when temporal coincidence existed between data from Darwin and Wolf. Datasets from Wolf’s anchorage at 20 m depth of 15-3-2016 to 19-10-2017 and from Wellington Reef at Darwin from 16 m depth of 17-3-2016 to 23-4-2017, as well as from Wellington Reef at 15m from 22-4-2017 to 4-4-2018 were compared (Fig 3).

**Fig 4:**
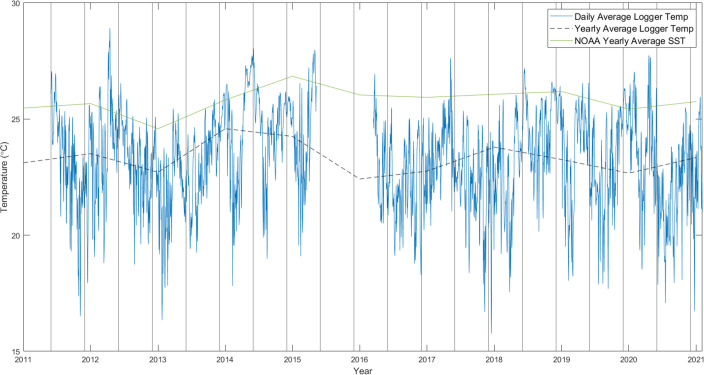
Yearly and daily temperature average from Wolf Island. Graph comparing the yearly (black dashed) and daily (blue) average temperature data from loggers at 20 m depth at the anchorage point of Wolf Island to the yearly average of NOAA SST data (NOAA, 2000) within the area of 1°, 2° latitude, and -92.5°, -91.5° longitude (green).

In order to verify the accuracy of the logger data values and their trends, data were compared to the NOAA Twice-Weekly Global 50km Satellite Coral Reef Watch (CRW) Sea Surface Temperature (SST) data [44, 45] between 2011 and 2019 (Fig 4) in an area of interest around the islands of Darwin and Wolf, between the 1° and 2° latitude, and -92.5° and -91.5° longitude, which equated to 4 pixels of temperature data (0.5° resolution of dataset). Due to these satellite monitoring products being retired in 2020, the new Daily Global 5 km Satellite Coral Bleaching Heat Stress Monitoring data was used for 2020 and 2021 [46]. This data was used to compare the average of the sea temperature for both islands and the surrounding water to the logger data. The SST data was then averaged across each year to produce a general trendline which could more easily be compared to the logger data. Since 50 km satellite products were retired in April 2020, data from 2020 were not used since the average would only include the warm season, leading to a positive bias in the data.

Temperatures at Darwin Island’s Wellington Reef mostly followed the same trend as at Wolf’s anchorage point, albeit at a higher temperature due to different depths between the loggers 16 m at Darwin, 20 m at Wolf (Fig 3). The main deviation between the datasets occurred from February to May 2017, where temperatures at Wellington Reef were consistently higher by 3–4°C, even reaching a difference of 7.45°C on 7-2-2017. Warming persisted at Darwin whilst temperatures were cooling at Wolf. Despite these discrepancies, the overall temperature ranges and trends were considered sufficiently similar to permit the assumption that the temperature data from Wolf’s anchorage point could be used as a useful indicator for the conditions at Darwin’s Wellington Reef.

The yearly average NOAA SST data (Fig 4) mostly followed the same trends as the 20 m logger, although the SST data was warmer. This was caused by the Wolf sensor being situated at 20 m depth, while the NOAA data modelled the ocean’s surface skin. The greater difference in 2016 can be partially explained by the logger data not beginning until midway through March. This caused the yearly average data point to miss over 3 months of warm season temperatures, lowering the average. The higher temperatures recorded by the Darwin logger in 2017 (Fig 3) can also be seen by the 3.2°C difference in the yearly average difference. Overall, this graph supports that the logger data from Wolf represent a useful trend to follow when analysing the Darwin *Caulerpa sp*. and hermatypic coral data, particularly since the site is located at a similar depth to the Wolf loggers.

## Results

### Subtidal ecological monitoring data

**Fig 5:**
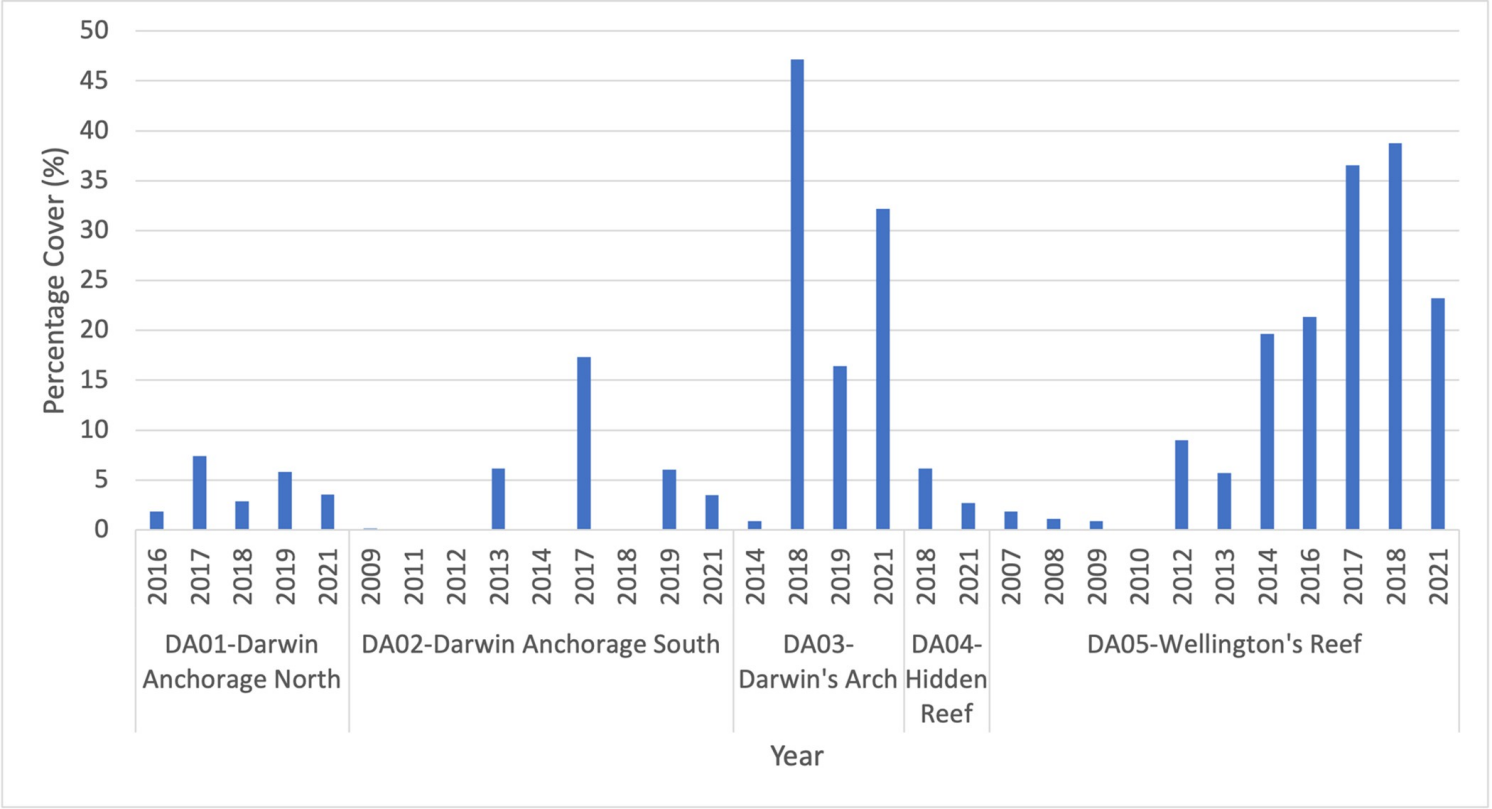
Percentage cover of *Caulerpa chemnitzia* in Darwin Island. Percentage cover of *Caulerpa chemnitzia* across the 5 sites studied in Darwin during the ecological monitoring project. All years included are those in which the site was monitored fully at least once during the year.

During 2007, coverage of C. chemnitzia was 1.85% (Fig 5) across DA05 and remained around the 1% mark for the following 2 years, when it also appeared at DA02. It was then absent in transects in 2010 and 2011. It may have persisted on the reef without being present along transects due to low abundance. In 2012 algae was detected exclusively at DA05 with a cover of 9.01%. In 2014 algae was found for the first time at Darwin’s Arch and increased sharply in cover at DA05 to 19.63%. The first appearance at Darwin Anchorage North (DA01) was in 2016, whilst cover at DA05 increased to 1.73%. Only DA01 and DA05 were monitored in 2016, therefore it is possible that cover of *C*. *chemnitzia* in DA02 and DA03 continued to increase as well. 2017 showed a strong increase across the sites for *C*. *chemnitzia*. The highest recorded coverage across the sites was in 2018, with the exception of the Darwin Anchorage Points (DA01 and DA02) which saw a reduction and complete absence respectively. During this year the algae was most abundant at the Arch with 47.16% of all quadrats covered as well as colonising the Hidden Reef (DA04) for the first recorded time. By March 2019 the cover at DA03 had declined sharply by 40.7%, whilst at Darwin Anchorage North (DA01) it increased slightly, and it reappeared at the South site (DA02). At the close of 2021, Caulerpa levels across the sites had decreased significantly, apart from Darwin’s Arch where it rose again to 32.16%.

The earliest recorded evidence of *Caulerpa chemnitzia* in Darwin according to the Subtidal Ecological Monitoring records was at Wellington Reef in 2007 with a percentage cover of just 1.85%. Up to this point only sites DA01 to DA03 had been monitored yearly since 2000, therefore it is possible there was some *C*. *chemnitzia* present at the reef before the first record. After the initial detection, percentage cover of the algae at the reef remained below this level, even disappearing from the transect in 2010, before increasing to 9.01% in 2012. Proceeding this there was a small dip in cover in 2013, but this was overshadowed by a continual sharp increase which extended to the year 2017, at which point the percentage cover plateaued around 38%. Unfortunately, there is no valid data available for the 2019–2020 period due to a changed transect location, which lead to a recorded cover of just 1.23% suggesting the algal spread is far from uniform across the reef, and COVID-19 complications. In 2021 the site was able to be monitored 3 separate times, producing an average cover of 23.2%. The second location to record the presence of *Caulerpa chemnitzia* in Darwin was the Southern Anchorage Point (DA02) in 2009, with only two of the possible 81 quadrant points falling on the algae and only in one quadrant. It then proceeded to disappear from recorded transects at the site until 2013 when it covered 6.17% of the transect, pointing to very low coverage at the site in the intermediate period. Its absence was once again noted in 2014, but it then rose to 17.35% cover by 2017, similar to the steep increase seen at Wellington Reef around the same time. From then on it has steadily decreased to 3.46%, barring an anomalous empty year in 2018. The Northern Anchorage Point (DA01) follows a steadier pattern than its sister site (DA02), with the first records of *C*. *chemnitzia* appearing in 2016, followed by its largest increase in 2017 of 5.56%, accompanying the general trend. It has since risen and fallen, with the current cover observed at 3.55%. The Hidden Reef site (DA04) only showed signs of *C*. *chemnitzia* cover for the first time in 2018 with 6.17%, although it had not been monitored in the previous three years (S1 Table), so was possibly colonised during the 2017 boom. Darwin’s Arch (DA03) appears to be the most volatile and is currently the site with the highest percentage cover of the algae around the island (32.16%). In addition, it also presented the highest levels of Caulerpa cover at Darwin overall, reaching 47.16% in 2018, which could possibly have been higher during the 2017 rise but unfortunately the site was not monitored that year. This site is consistently the most disturbed due to recreational diving, which may have an effect on the ability of the algae to take a foothold and spread.

### Photo-transect data

**Fig 6:**
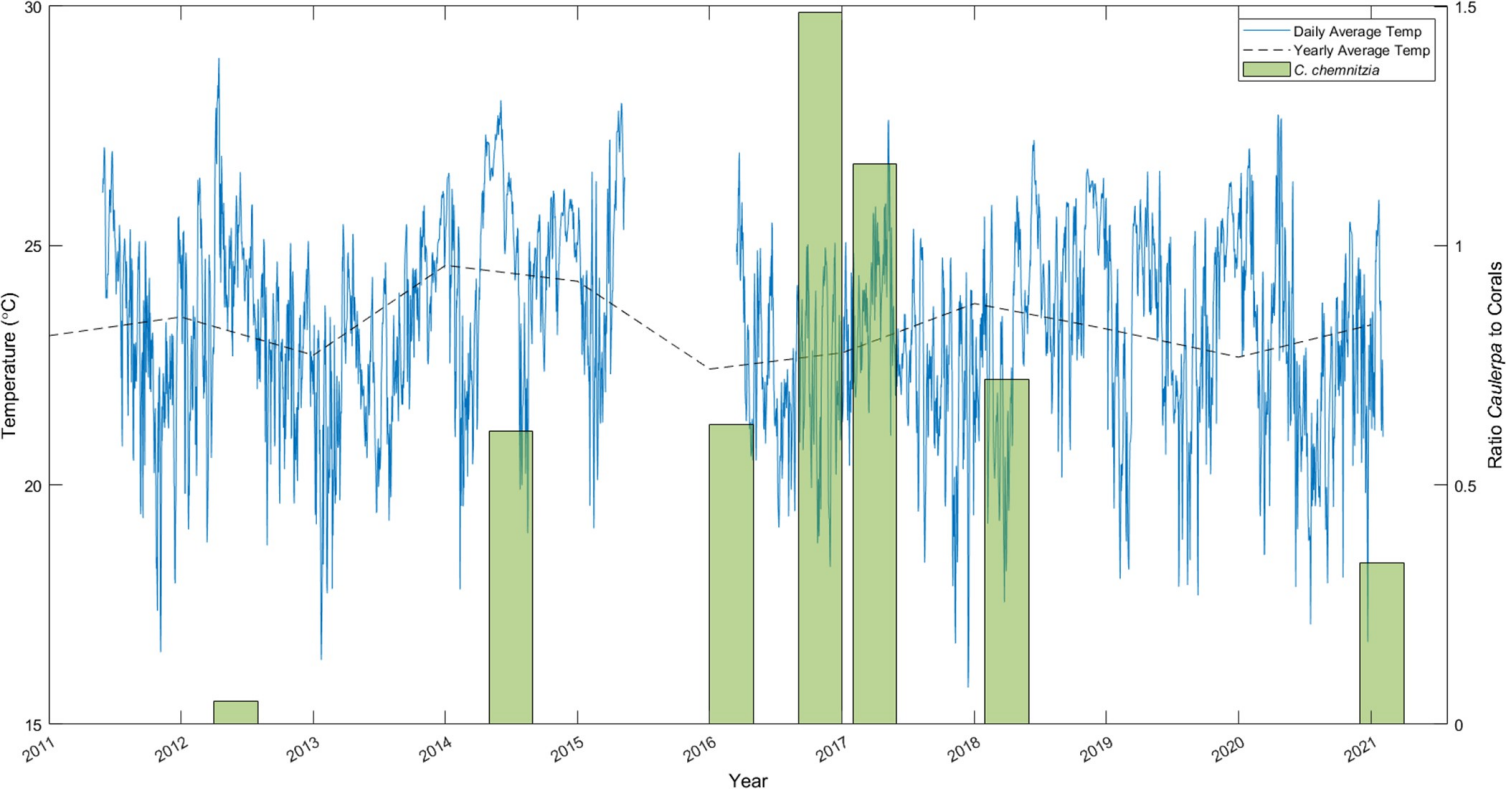
*Caulerpa chemnitzia* cover at Wellington reef. Graph showing the ratio of C. chemnitzia cover to hermatypic coral cover (green bar) at Wellington reef between 2012 and 2021, with the yearly (black dash) and daily (blue line) averaged 20 m logger temperatures at the Wolf Island anchorage point.

In June 2012, *Caulerpa* was barely present, with at total coverage of just 1.7% across the transect and coral cover at twenty-one times that of the algae. By July of 2014, algae cover had increased to 22.6% and coral cover was only 1.6 times that of algae cover. From October 2014, a strong ENSO event lasted a year and a half, with SST anomalies reaching +2.6°C [16]. Algae cover continued to increase to 29.6% by March 2016, but at a slower rate. Coral cover remained high and the ratio *Caulerpa* to corals changed little. Coral coverage was not greatly affected by the ENSO due to the persistence of upwelling events occurring at the same time which kept temperatures from rising to bleaching levels [33]. The ENSO was reflected in higher yearly temperature averages (except for 2016 due to missing data) and less extreme temperature troughs (Fig 4). In November 2016 the photo-transects contained little coral coverage and algae reached a peak of 39.64%, causing a sharp spike in algae-coral ratio (Fig 6). This was accompanied by a fall in the coral cover recorded at the time, possibly caused by variations in the transect location. High algae cover continued five months later with cover but no longer increased, dropping to 38.8%. Algae were possibly aided by continuously high temperatures, logged at 16 m depth at Wellington Reef from February-May 2017 (Fig 3). Also, coral cover increased. Towards the end of 2017 and early 2018, a La Niña caused a maximum negative anomaly of 1.0°C across the tropical Pacific [16]. Nevertheless, consistently higher yearly average temperatures have been maintained since 2014 (S1 Fig). This drop in temperature was followed by a steep fall in the percentage cover of *C*. *chemnitzia* at Wellington Reef by April 2018, down to 29.1%, while coral cover kept increasing. Algae cover continued to fall until it reached 15.2% in February 2021 (Fig 6).

## Discussion

This study shows a succession of rapid increase followed by a decrease in abundance of the alga *Caulerpa chemnitzia* at Wellington Reef on Darwin Island, the only framework coral reef in the Galapagos Archipelago [41]. Prior to the outbreak of *C*. *chemitziae*, another alga, *Peysonnellia boergesenii*, was from 2007–2012 the most common species competing with corals and causing partial mortality on Wellington Reef [33]. From 2012, *C*. *chemnitzia* began first occupying non-coral substrate and then competing with corals by overgrowing them and smothering the covered tissues. Approximately 40% of free substrate was covered by this species [33]. Understanding the dynamics of *C*. *chemnitzia* on this important reef system is vital. Unhindered colonization of free substratum and increased overgrowth and competition with corals may change the dynamics of Darwin benthic ecosystems and may result in profound habitat alterations on the only framework coral reef of the Galapagos [47].

This alga increased in abundance and overgrew corals on the reef since 2012, ultimately raising fears that a phase-shift from coral to algae [4, 48–51] might be imminent. However, from 2019 onwards algae populations strongly contracted and while not having returned to baseline level, there is currently low risk of corals being displaced. Prior to this highly unusual outbreak, no reports existed of this species being present. During the Pacific Expeditions in the 1930’s Taylor reported *C*. *peltata*, potentially a misidentification of *C*. *chemnitzia*, from nearby Wolf Island (Fig 1B). Since the expedition did not visit Darwin Island [21], it is unknown whether this species was present in Darwin at that time. *C*. *chemnitzia* is the most common and widespread species of *Caulerpa* in the ETP with a distribution along the coast from Mexico to Ecuador including the iconic MPAs of Ravillagigedo National Park (Mexico), Clipperton Atoll (France), Gorgona National Natural Park (Colombia) and Galapagos Marine Reserve (Ecuador) [32], It remains unclear when and how this species arrived at Darwin, however, it can be hypothesised that due to the dynamic surface current regimen in the ETP *C*. *chemnitzia* propagules, mainly vegetative fragments could have been transported by the Panama Current from any of the above-mentioned coasts or MPAs.

Furthermore, the Galapagos Archipelago has received vessels from around the world since its discovery in 1535. This long history of marine traffic has facilitated introduction of alien species [7] and as tourism, trade and transport increase due to local and global growth, the amount of marine traffic that enters the Galapagos Marine Reserve (GMR) and navigates between islands has increased as well [52]. Fouling of ship hulls is a common potential vector for introductions of macroalgal species [53, 54] and could be the source of the introduction of this species to Wellington Reef. The authors cannot confirm how this species arrived at Wellington reef, which is why *C*. *chemnitzia* has been classed as a cryptogenic species in the GMR until more information is available [7].

*Caulerpa chemitzia* has been registered at seven GMR locations including Wolf Island with the highest abundance at Wellington Reef (I. Keith, personal communication, May 11, 2022). In other sites, this species existed in small patches that did not form extensive mats covering the substrate. Sites were located in protected bays at shallower depths (1–6 m) than at Wellington Reef that lies between 12–18 m and is exposed to strong currents especially from June to December. It remains unclear why *C*. *chemnitzia* reached such high abundances on this exposed reef system in the far north region of the archipelago, however, there are several factors surrounding this system that can be explored to better understand this species expansion, including temperature and changes in upwelling and nutrient levels.

Wellington Reef is characterized by large colonies of corals mainly *Porites lobata*, *Pavona clavus* and *Pocillopora spp* [9]. The reef is influenced by the nutrient-poor warm waters of the Panama Current that flows north to south towards the Galapagos Islands reducing upwellings in the area. The sea surface temperature (HadISST, 1970–2018) at Darwin shows an average of 26.28°C and in fact studies have illustrated that compared to other regions in the archipelago including the Island of Wolf, Darwin has the lowest upwelling index during the sampled periods, and this coincides with lower nutrient levels than the rest of the archipelago [9]. The authors hypothesize that this species could have expanded its distribution over Wellington Reef because of its known morphological plasticity [32, 36, 55] due to a response to change in the environment, in this case high temperature and low nutrients. This species could have used this morphological plasticity giving the specie the opportunity to change behaviour and expand over the reef. This change in behaviour could be considered as invasive due to the quick and aggressive manner it behaved, however the data shows a decline in abundance after a short time leaving the authors to rather consider this event as a phase shift caused by the changing environmental conditions in the region.

Warming-related phase shifts in marine systems can cause successive stages of geographic extension or contraction of species ranges [56]. The Galapagos Islands are among the most vulnerable sites in the ETP due to potential impacts of climate change and because they are regularly subjected to climate variability by El Niño Southern Oscillation (ENSO) events. During ENSO, prolonged increases in sea temperature are induced, as the warm surface waters of the western Pacific band migrate to the coast of South America which may open avenues for non-native species to reach the islands [37]. Prior to the 1982/83 El Niño Southern Oscillation (ENSO) coral habitats in the Galapagos were made up of low diversity coral communities and small coral reefs which were actively accreting [57]. This ENSO event brought with it disastrous impacts for both local wildlife causing several extinctions [9, 28, 31] and coral reefs across the islands, recording an average of 97% mortality of the latter as well as significant bleaching among the remaining coral populations. In fact, of the 17 original coral reefs documented by Glynn the only one that remained after this event is Wellington Reef. ENSO events are predicted to increase in intensity and frequency due to the impact of climate change, which in turn may cause the deterioration of marine ecosystems [5, 8, 33]. The recovery of these ecosystems will depend on the length of intervals between strong climatic impacts and will be dependent upon the life history and biology. Shifts in species composition due to shifts in temperature and productivity can generate a different ecological equilibrium, with different biodiversity and functional state, altering the susceptibility of the Islands to invasions.

A surprising number of marine species have invaded marine protected areas, including Galapagos [7] and may threaten or diminish their high conservation and social value. Preventing the introduction of non-native species through biosecurity is the most cost-effective strategy, rather than managing them once they become established. Thus, effective biosecurity systems are required to minimize the risk of invasive species introductions and a functional alert system with Early Detection Rapid Response (EDRR) protocols needs to be implemented in the Galapagos Biosecurity program to avoid climate driven Non-Indigenous Species (NIS) entering the GMR or for native species that become invasive due to warming-related phase shifts. Controlling NIS arising from climate change is of high importance to safeguard the marine ecosystems of the Galapagos. Investing now in the precautionary principle will allow for the adaptation mechanism to be more effective and more cost-efficient.

## Supporting information

S1 Fig Graph showing the yearly averaged NOAA SST data from the Coral Reed Watch for the region surrounding Darwin and Wolf (lon: (-92.5, -91.5), lat: (1, 2)). Data is at 50 km resolution from 2001 to 2019 and at 5 km resolution from 2020 onwards.
(DOCX)

S1 Table Table showing the sites monitored at Darwin since the beginning of the subtidal ecological monitoring project in 2004. Sites listed with a tick were monitored at least once during the year, those marked with a P are occasions on which the divers could not do a full monitoring of the site and only registered presence of species rather than percentage cover. The site marked with an “In” represents an inconsistent transect which was placed in a different location to normal and whose data is incompatible with the rest.
(DOCX)

S1 File MATLAB script. (DOCX)
