# Supplementary material for: Caulerpa chemnitzia in Darwin threatening Galapagos coral reefs
Source: PLoS One. 2022 Aug 31;17(8):e0272581. doi: 10.1371/journal.pone.0272581 (PMC9432695; doi:10.1371/journal.pone.0272581)
Supplement: S1 Fig — Data is at 50 km resolution from 2001 to 2019 and at 5 km resolution from 2020 onwards. (DOCX) [file pone.0272581.s001.docx]

## **Supporting information**


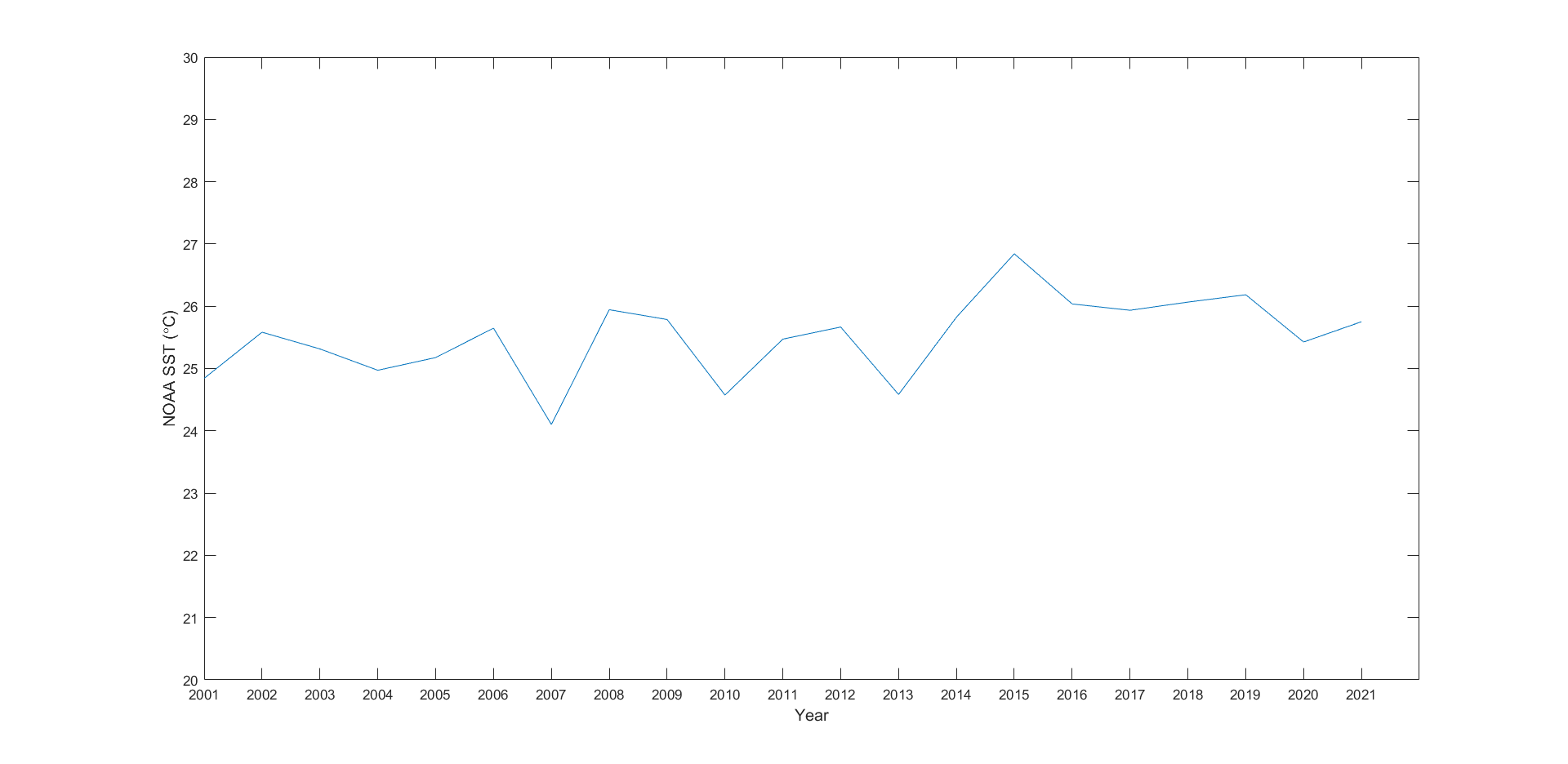


S1 Fig. Graph showing the yearly averaged NOAA SST data from the Coral Reed Watch for the region surrounding Darwin and Wolf (lon: (-92.5, -91.5), lat: (1, 2)). Data is at 50 km resolution from 2001 to 2019 and at 5 km resolution from 2020 onwards.
