# Supplementary material for: Caulerpa chemnitzia in Darwin threatening Galapagos coral reefs
Source: PLoS One. 2022 Aug 31;17(8):e0272581. doi: 10.1371/journal.pone.0272581 (PMC9432695; doi:10.1371/journal.pone.0272581)
Supplement: S1 Table — Sites listed with a tick were monitored at least once during the year, those marked with a P are occasions on which the divers could not do a full monitoring of the site and only registered presence of species rather than percentage cover. The site marked with an “In” represents an inconsistent transect which was placed in a different location to normal and whose data is incompatible with the rest. (DOCX) [file pone.0272581.s002.docx]

S1 Table. Table showing the sites monitored at Darwin since the beginning of the Subtidal Ecological Monitoring Project in 2004. Sites listed with a tick were monitored at least once during the year, those marked with a P are occasions on which the divers could not do a full monitoring of the site and only registered presence of species rather than percentage cover. The site marked with an “In” represents an inconsistent transect which was placed in a different location to normal and whose data is incompatible with the rest.

|  | DA01 | DA02 | DA03 | DA04 | DA05 |
| --- | --- | --- | --- | --- | --- |
| 2004 |  | ✓ | ✓ |  |  |
| 2005 | ✓ | ✓ | ✓ |  |  |
| 2006 | ✓ | ✓ | ✓ |  |  |
| 2007 | ✓ | ✓ | ✓ |  | ✓ |
| 2008 | ✓ | ✓ | ✓ | ✓ | ✓ |
| 2009 | ✓ | ✓ |  | ✓ | ✓ |
| 2010 | ✓ | P | ✓ | ✓ | ✓ |
| 2011 | ✓ | ✓ | P | ✓ | P |
| 2012 | ✓ | ✓ | ✓ | ✓ | ✓ |
| 2013 | ✓ | ✓ | ✓ |  | ✓ |
| 2015 |  |  |  |  |  |
| 2014 | ✓ | ✓ | ✓ | ✓ | ✓ |
| 2016 | ✓ |  |  |  | ✓ |
| 2017 | ✓ | ✓ |  |  | ✓ |
| 2018 | ✓ | ✓ | ✓ | ✓ | ✓ |
| 2019 | ✓ | ✓ | ✓ |  | In |
| 2021 | ✓ | ✓ | ✓ | ✓ | ✓ |
