# Supplementary material for: Caulerpa chemnitzia in Darwin threatening Galapagos coral reefs
Source: PLoS One. 2022 Aug 31;17(8):e0272581. doi: 10.1371/journal.pone.0272581 (PMC9432695; doi:10.1371/journal.pone.0272581)
Supplement: S1 File — (DOCX) [file pone.0272581.s003.docx]

## S1 File. Scripts

Overall graphs:

%% Wolf Fondeadero 20m 2011 to 2015

t1=datetime(2011,5,29,17,30,0);

t2=datetime(2012,5,26,6,30,0);

t3=t1:minutes(30):t2;

DF20_1=t3';

t1=datetime(2012,5,27,11,30,0);

t2=datetime(2013,5,8,16,0,0);

t3=t1:minutes(30):t2;

DF20_2=t3';

t1=datetime(2013,5,8,16,30,0);

t2=datetime(2015,5,11,16,30,0);

t3=t1:minutes(30):t2;

DF20_3=t3';

DF20=[DF20_1;DF20_2;DF20_3];

TF20=xlsread('Wolf_Fondeadero_1115_20m.xlsx','B1:B69206');

%Timetable

mytab20=table(DF20,TF20);

% Convert to timetable

TT20 = table2timetable(mytab20);

% Calculate average value per day

DailyAvg20 = retime(TT20,'daily','mean');

MontAvg20=retime(TT20,'weekly','mean');

YearAvg20=retime(TT20,'yearly','mean');

tplot20=DailyAvg20.DF20;

tmplot20=MontAvg20.DF20;

%typlot20=YearAvg20.DF20;

templot20=DailyAvg20.TF20;

temmplot20=MontAvg20.TF20;

%temyplot20=YearAvg20.TF20;

%% Wolf Fondeadero 15m 2016 to 2017

%changed from GMT-4 to GMT-6

t1=datetime(2016,03,15,23,0,0);

t2=datetime(2017,04,28,15,0,0);

t3=t1:hours(1):t2;

DF15=t3';

TF15=xlsread('Wolf_Fondeadero_15m_1617.csv','C4:C9812');

%Timetable

mytab15=table(DF15,TF15);

% Convert to timetable

TT15 = table2timetable(mytab15);

% Calculate average value per day

DailyAvg15 = retime(TT15,'daily','mean');

MontAvg15=retime(TT15,'weekly','mean');

YearAvg15=retime(TT15,'yearly','mean');

tplot15=DailyAvg15.DF15;

tmplot15=MontAvg15.DF15;

%typlot15=YearAvg15.DF15;

templot15=DailyAvg15.TF15;

temmplot15=MontAvg15.TF15;

%temyplot15=YearAvg15.TF15;

%% Wolf Fondeadero 20m 2017 to 2021

t1=datetime(2017,04,28,15,30,0);

t2=datetime(2018,06,16,15,30,0);

t3=t1:minutes(30):t2;

DF20_1=t3';

t1=datetime(2018,06,16,15,49,41);

t2=datetime(2019,03,21,15,19,41);

t3=t1:minutes(30):t2;

DF20_2=t3';

t1=datetime(2019,03,21,16,0,0);

t2=datetime(2021,02,03,15,0,0);

t3=t1:minutes(30):t2;

DF20_3=t3';

DF20B=[DF20_1;DF20_2;DF20_3];

TF20B=xlsread('Wolf_Fondeadero_1721_20m.xlsx','B1:B66096');

%Timetable

mytab20B=table(DF20B,TF20B);

% Convert to timetable

TT20B = table2timetable(mytab20B);

% Calculate average value per day

DailyAvg20B = retime(TT20B,'daily','mean');

MontAvg20B=retime(TT20B,'weekly','mean');

YearAvg20B=retime(TT20B,'yearly','mean');

tplot20B=DailyAvg20B.DF20B;

tmplot20B=MontAvg20B.DF20B;

%typlot20B=YearAvg20B.DF20B;

templot20B=DailyAvg20B.TF20B;

temmplot20B=MontAvg20B.TF20B;

%temyplot20B=YearAvg20B.TF20B;

%% Temp means

%temp1=mean(templot10);

temp2=mean(templot15);

temp3=mean(templot20);

temp3b=mean(templot20B);

temp_av=mean([temp2,temp3,temp3b]);

%% Temp concats

templotfin=[templot20;templot15;templot20B];

tplotfin=[tplot20;tplot15;tplot20B];

%Mean

%Timetable

mytabfin=table(tplotfin,templotfin);

% Convert to timetable

TTF = table2timetable(mytabfin);

MontAvg=retime(TTF,'monthly','mean');

YearAvg=retime(TTF,'yearly','mean');

tmplotfina=MontAvg.tplotfin;

typlotfina=YearAvg.tplotfin;

temmplotfina=MontAvg.templotfin;

temyplotfina=YearAvg.templotfin;

%Variance

MontVar=retime(TTF,'monthly',@var);

YearVar=retime(TTF,'yearly',@var);

tmplotfinv=MontVar.tplotfin;

typlotfinv=YearVar.tplotfin;

temmplotfinv=MontVar.templotfin;

temyplotfinv=YearVar.templotfin;

%Range

YearRan=retime(TTF,'yearly',@range);

typlotfinr=YearRan.tplotfin;

temyplotfinr=YearRan.templotfin;

%% Caulerpa/Corals data

[cau,~,~]=xlsread('Combined_charts.xlsx','Sheet1','C1:C8');

[cor,~,~]=xlsread('Combined_charts.xlsx','Sheet1','B1:B8');

date=[datetime(2012,6,1,0,0,0);datetime(2014,7,1,0,0,0);datetime(2016,3,1,0,0,0);datetime(2016,11,1,0,0,0);datetime(2017,4,1,0,0,0);datetime(2018,4,1,0,0,0);datetime(2021,2,1,0,0,0)];

d_plot=datenum(date);

com=[cau,cor];

%t=table(d_plot,cau,cor);

%% Cau Cor

cordiv=1./cor;

cor1=cordiv.*cor;

cau1=cau.*cordiv;

caucor=[cau1,cor1];

%% Overall plot

time_labs=[datetime(2011,1,1,0,0,0);datetime(2012,1,1,0,0,0);datetime(2013,1,1,0,0,0);...

datetime(2014,1,1,0,0,0);datetime(2015,1,1,0,0,0);datetime(2016,1,1,0,0,0);...

datetime(2017,1,1,0,0,0);datetime(2018,1,1,0,0,0);datetime(2019,1,1,0,0,0);...

datetime(2020,1,1,0,0,0);datetime(2021,1,1,0,0,0);];

time_labs2=[datetime(2011,1,1,0,0,0);datetime(2012,1,1,0,0,0);date(1);datetime(2013,1,1,0,0,0);...

datetime(2014,1,1,0,0,0);date(2);datetime(2015,1,1,0,0,0);datetime(2016,1,1,0,0,0);date(3);...

date(4);datetime(2017,1,1,0,0,0);date(5);datetime(2018,1,1,0,0,0);date(6);datetime(2019,1,1,0,0,0);...

datetime(2020,1,1,0,0,0);datetime(2021,1,1,0,0,0);date(7)];

figure

yyaxis left

plot(tplot20,templot20,'-','Color',[0 0.4470 0.7410],'HandleVisibility','off')

hold on

plot(tplot20B,templot20B,'-','Color',[0 0.4470 0.7410],'HandleVisibility','off')

plot(tplot15,templot15,'-','Color',[0 0.4470 0.7410])

%plot(tmplotfina,temmplotfina,'-b')

plot(typlotfina,temyplotfina,'--k')

hold off

ylim([15 30])

ylabel('Temperature (\circC)')

yyaxis right

b=bar(date,caucor);

b(1).FaceColor=[0.4660 0.6740 0.1880];

b(2).FaceColor=[0.8500 0.3250 0.0980];

legend('Daily Average Temp','Yearly Average Temp','Caulerpa sp.','Hermatypic Corals')

ylabel('Ratio')

xlabel('Year')

xticks(time_labs)

%% Variance plot

figure

yyaxis left

plot(tplot20,templot20,'-','Color',[0 0.4470 0.7410],'HandleVisibility','off')

hold on

plot(tplot20B,templot20B,'-','Color',[0 0.4470 0.7410],'HandleVisibility','off')

plot(tplot15,templot15,'-','Color',[0 0.4470 0.7410])

plot(tmplotfinv,temmplotfinv,'-.r')

plot(typlotfinv,temyplotfinv,'--k')

hold off

ylabel('Temperature (\circC)')

yyaxis right

b=bar(date,caucor);

b(1).FaceColor=[0.4660 0.6740 0.1880];

b(2).FaceColor=[0.8500 0.3250 0.0980];

ylabel('Ratio')

xlabel('Year')

legend('Daily Average Temp','Monthly Temp Variance','Yearly Temp Variance','Caulerpa sp.','Hermatypic Corals')

xticks(time_labs)

%% Range Plot

figure

yyaxis left

plot(tplot20,templot20,'-','Color',[0 0.4470 0.7410],'HandleVisibility','off')

hold on

plot(tplot20B,templot20B,'-','Color',[0 0.4470 0.7410],'HandleVisibility','off')

plot(tplot15,templot15,'-','Color',[0 0.4470 0.7410])

plot(typlotfinr,temyplotfinr,'--k')

hold off

ylabel('Temperature (\circC)')

yyaxis right

b=bar(date,caucor);

b(1).FaceColor=[0.4660 0.6740 0.1880];

b(2).FaceColor=[0.8500 0.3250 0.0980];

ylabel('Ratio')

xlabel('Year')

legend('Daily Average Temp','Yearly Temp Range','Caulerpa sp.','Hermatypic Corals')

xticks(time_labs)

%% Warm/Cold season

t1=datetime(2011,06,01,0,0,0);

t2=datetime(2020,12,01,0,0,0);

sea_lins=t1:calmonths(6):t2;

figure

plot(tmplot20,temmplot20,'-','Color',[0 0.4470 0.7410],'HandleVisibility','off')

hold on

plot(tmplot20B,temmplot20B,'-','Color',[0 0.4470 0.7410],'HandleVisibility','off')

plot(tmplot15,temmplot15,'-','Color',[0 0.4470 0.7410])

for i=1:length(sea_lins)

xline([sea_lins(i)])

end

hold off

ylabel('Temperature (\circ)')

xlabel('Year')

ylim([17 28])

%% Overall plot 2

figure

yyaxis left

plot(tplot20,templot20,'-','Color',[0 0.4470 0.7410],'HandleVisibility','off')

hold on

plot(tplot20B,templot20B,'-','Color',[0 0.4470 0.7410],'HandleVisibility','off')

plot(tplot15,templot15,'-','Color',[0 0.4470 0.7410])

%plot(tmplotfina,temmplotfina,'-b')

plot(typlotfina,temyplotfina,'--k')

hold off

ylim([15 30])

ylabel('Temperature (\circC)','FontSize',12,'Color','k')

set(gca,'YColor','k');

yyaxis right

b=bar(date,cau1);

b.FaceColor=[0.4660 0.6740 0.1880];

b.FaceAlpha=0.5;

legend('Daily Average Temp','Yearly Average Temp','\itC. chemnitzia')

ylabel('Ratio {\itCaulerpa} to Corals','FontSize',12,'Color','k')

xlabel('Year','FontSize',12,'Color','k')

set(gca,'YColor','k');

xticks(time_labs)

NOAA data script

%% Data extraction of monthly averages

%LonLat for DW: (-92.5,1) (-91.5,2), lat(i): 167:169, lon(i): 176:178

year=[2001:2021];

lat=hdfread('crw_oper50km_monthlymean_sst_201701.hdf','latitude','index',{[167],[],[3]});

lon=hdfread('crw_oper50km_monthlymean_sst_201701.hdf','longitude','index',{[176],[],[3]});

hin=hdfinfo('crw_oper50km_monthlymean_sst_201701.hdf');

for k=1:17

if k>=1 && k<=9

for i=1:9

%info=hdfinfo(sprintf('crw_oper50km_monthlymean_sst_20170%d.hdf',i));

tdata(k,i,:,:)=0.01*hdfread(sprintf('crw_oper50km_monthlymean_sst_200%d0%d.hdf',k,i)...

,'CRW_SST','index',{[167 176],[],[3 3]});

end

for i=10:12

tdata(k,i,:,:)=0.01*hdfread(sprintf('crw_oper50km_monthlymean_sst_200%d%d.hdf',k,i)...

,'CRW_SST','index',{[167 176],[],[3 3]});

end

elseif k>=10

for i=1:9

tdata(k,i,:,:)=0.01*hdfread(sprintf('crw_oper50km_monthlymean_sst_20%d0%d.hdf',k,i)...

,'CRW_SST','index',{[167 176],[],[3 3]});

end

for i=10:12

tdata(k,i,:,:)=0.01*hdfread(sprintf('crw_oper50km_monthlymean_sst_20%d%d.hdf',k,i)...

,'CRW_SST','index',{[167 176],[],[3 3]});

end

end

end

k=17;

for j=1:2

k=k+1;

myfolder=sprintf('D:\\Caul_Cor\\NOAA data\\20%d',k);

filepattern=fullfile(myfolder, '*.hdf');

files = dir(filepattern);

%baseFileName = files(1).name;

for i=1:length(files)

if k==18

t18(i,:,:)=0.01*hdfread(files(i).name,'CRW_SST','index',{[167 176],[],[3 3]});

elseif k==19

t19(i,:,:)=0.01*hdfread(files(i).name,'CRW_SST','index',{[167 176],[],[3 3]});

for jj=1:2

k=k+1;

myfolder=sprintf('D:\\Caul_Cor\\NOAA data\\5km\\20%d',k);

filepattern=fullfile(myfolder, '*.nc');

files = dir(filepattern);

for i=1:length(files)

if k==20

t20(i,:,:)=nc_varget(files(i).name,'analysed_sst',[0 1820 1750],[1 20 21]);

elseif k==21

t21(i,:,:)=nc_varget(files(i).name,'analysed_sst',[0 1820 1750],[1 20 21]);

end

end

end

%% Yearly Averages

for i=1:length(t18)

t_squ=squeeze(t18(i,:,:));

t_calc(i)=mean(t_squ,'all');

end

t18_av=mean(t_calc);

clearvars t_calc

for i=1:length(t19)

t_squ=squeeze(t19(i,:,:));

t_calc(i)=mean(t_squ,'all');

end

t19_av=mean(t_calc);

clearvars t_calc

for i=1:length(t20)

t_squ=squeeze(t20(i,:,:));

t_calc(i)=mean(t_squ,'all');

end

t20_av=mean(t_calc);

clearvars t_calc

for i=1:length(t21)

t_squ=squeeze(t21(i,:,:));

t_calc(i)=mean(t_squ,'all');

end

t21_av=mean(t_calc);

clearvars t_calc

tend=[t18_av,t19_av,t20_av,t21_av];

for i=1:17

for j=1:12

t_sq=squeeze(tdata(i,j,:,:));

t_calc(j)=mean(t_sq,'all');

end

t_yav1(i)=mean(t_calc);

clearvars t_calc

end

t_yav=[t_yav1,tend];

time_labs=[datetime(2011,1,1,0,0,0);datetime(2012,1,1,0,0,0);datetime(2013,1,1,0,0,0);...

datetime(2014,1,1,0,0,0);datetime(2015,1,1,0,0,0);datetime(2016,1,1,0,0,0);...

datetime(2017,1,1,0,0,0);datetime(2018,1,1,0,0,0);datetime(2019,1,1,0,0,0);...

datetime(2020,1,1,0,0,0);datetime(2021,1,1,0,0,0)];

%% Figures

figure

plot(year,t_yav)

ylim([20 30])

xlim([2001 2022])

xlabel('Year','FontSize',12)

ylabel('NOAA SST (\circC)','FontSize',12)

xticks([2001:1:2021])

figure

plot(tplot20,templot20,'-','Color',[0 0.4470 0.7410],'HandleVisibility','off')

hold on

plot(tplot20B,templot20B,'-','Color',[0 0.4470 0.7410],'HandleVisibility','off')

plot(tplot15,templot15,'-','Color',[0 0.4470 0.7410])

plot(typlotfina,temyplotfina,'--k')

plot(time_labs,t_yav(11:21))

for i=1:length(sea_lins)

xline([sea_lins(i)])

end

hold off

ylim([15 30])

ylabel('Temperature (\circC)','FontSize',12)

legend('Daily Average Logger Temp','Yearly Average Logger Temp','NOAA Yearly Average SST','FontSize',12)

xlabel('Year','FontSize',12)

xticks(time_labs)
